# A Simple and Precise Procedure for a Complete Characterization of a Cone-Beam Computed Tomography System

**DOI:** 10.3390/s25051282

**Published:** 2025-02-20

**Authors:** Kun-Long Shih, Shih-Chun Jin, Chia-Wei Wang, Jyh-Cheng Chen

**Affiliations:** 1 Department of Electro-Optical Engineering, National Taipei University of Technology, Taipei 106344, Taiwan; klshih@ntut.edu.tw (K.-L.S.); a6210647@ntut.edu.tw (S.-C.J.); 2 Department of Biomedical Imaging and Radiological Sciences, National Yang Ming Chiao Tung University, Taipei 112304, Taiwan; way84131.be12@nycu.edu.tw; 3 Department of Biomedical Imaging and Radiological Sciences, China Medical University, Taichung 404328, Taiwan; 4 School of Radiology, Shandong First Medical University and Shandong Academy of Medical Sciences, Taian 271000, China

**Keywords:** ellipse fitting, calibration, cone-beam computed tomography, precision alignment loop

## Abstract

In the calibration of cone-beam computed tomography (CBCT), two factors must be checked: the alignment of the imaging detector of the CBCT system and the effect of the slanted sample platform. Previously, we developed and validated a distinct procedure to accurately calibrate any misalignment of the detector by using a cylindrical phantom with beads in a straight line, parallel to the axis of rotation of the CBCT system. Here, we generalize our earlier procedure to calibrate the CBCT system while also detecting and rectifying a slight slant of the sample platform. We revise and validate our new procedure by calibrating the CBCT system, which also determines the tilt angle between the central axis of the phantom and the axis of rotation, when not 0°. The errors in misaligned angles for our new procedure are within ±0.03°, calibrating the CBCT system more precisely than our earlier work. To confirm this, we have performed a complete, precise calibration of a dental CBCT system with a tilting sample platform. We also reconstruct a HA phantom in this CBCT system to analyze the quality of reconstruction. We present here a validated method for calibrating a CBCT system and rectifying the effect of its tilting sample platform with good accuracy.

## 1. Introduction

Existing geometric calibrations rely on determining a set of parameters that accurately characterize the imaging system’s geometry, by setting the axis of rotation (AOR) perpendicular to the sample platform. The well-known calibration phantoms containing steel beads set in special patterns are used in most geometric calibration methods. The most extensively used phantom types include beads in circular [1,2,3], helical [4,5], linear [6,7,8,9,10], or other special arrangements [11,12].

Shih et al. [6] proposed and validated an analytical geometric scheme to calibrate, find, and correct any misalignment in the imaging detector relative to the X-ray source. Here, we modify that scheme to include rectifying images taken with a tilted sample platform (or holder), thereby calibrating the complete cone-beam computed tomography (CBCT) system, including the tilt of its sample platform. We validate this new scheme by using a helical-beads phantom and a line-beads phantom, allowing for a tilt between the AOR and the central axis of the phantom (AOP). Section 2.1 describes the dental CBCT geometry and phantoms of calibration. Section 2.2 will show how the beads in the two chosen phantoms can have varied rotational radii. The fundamental theorem of an ideal CBCT system, where the beads in the phantoms may have varied rotating radii, is defined in Section 2.3. The precise relationship between minor misalignment and perfect alignment are delineated in Section 2.4–these relationships provide the necessary variables to depict the misalignment and form the foundation of the new precision alignment loop (PAL), generalized from our earlier version [6]. Section 3.1 presents the criterion for validating PAL’s analytical simulations. Section 3.2 and Section 3.3 will validate the new PAL by detecting the misaligned parameter-set in the simulations for the helical-beads phantom (Section 3.2) and the line-beads phantom (Section 3.3), both having a slightly tilted AOP relative to the AOR. In our analytical simulations, we set the beads and the X-ray source as points, with each bead projected onto a misaligned detector by stretching a line from the source to the bead to intersect the misaligned detector. Section 4 presents the calibrating results of a dental CBCT system using our new scheme. 

C-arm CBCT offers CT-like 3D imaging capabilities while being appropriate for interventional suites. Usually, this system is pre-calibrated using a calibration phantom under the assumption that the C-arm motion is reproducible. However, the mechanical instabilities, scatter, and motion of patient account for the image artifacts and decreased contrast-to-noise (CNR) [13].

## 2. Materials and Methods

### 2.1. The Dental CBCT Geometry

**Figure 1:**
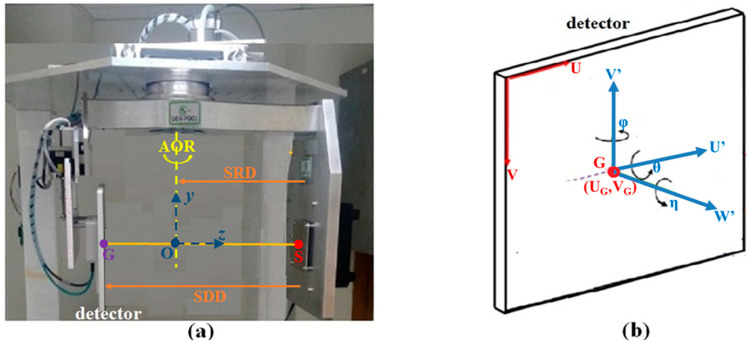
(**a**) The dental CBCT geometry. S represents the X-ray point source, O is on the axis of rotation, and G is the detector’s geometrical center. The x-axis points into the y-z plane, which is not shown. (**b**) (*θ*, *φ*, *η*) are the three tilt angles of the detector’s local *U*′-*V*′-*W*′ coordinate at G.

The dental CBCT system under study, shown in Figure 1a, has a flat detector and an X-ray source on opposite sides of a AOR, mounted on a rotating gantry. The central ray from the X-ray focal point S passes O on the AOR perpendicularly and intersects the detector at the geometric center G, or simply “the center G”. The three points, S, O, and G, lie on the system’s principal axis, the *z*-axis, originating from O pointing toward S. The *y*-axis is the AOR. The *x*-axis, with its origin at O, follows the rule of right-hand screw. The distance between S and O is the source-to-rotational-axis distance (SRD), and the distance between S and G is source-to-detector distance (SDD). The coordinate system that defines the detector’s orientation is illustrated in Figure 1b.

In an ideally aligned system, the *U*′-*V*′-*W*′ coordinate (a local system) is a translation of the *x*-*y*-z coordinate (the global system) along the *z*-axis to the detector. The position (*U_i_*, *V_j_*) of the (*i, j*)th pixel is given by the *U-V* system, the detector’s body coordinate, which is co-planar with the *U*′-*V*′ system. If the system is not aligned, then the misalignment (*θ*, *φ*, *η*) can be characterized by a rotation of the axes at the center G: slant angle *η* about the *W*′-axis, skew angle *φ* about the *V*′-axis, and tilt angle *θ* about the *U*′-axis. In a misaligned system, when θ or φ is not zero, the *z*-axis is not overlapping with the *W*’-axis. The set (*U_G_*, *V_G_*, *SRD*, *SDD*, *θ*, *φ*, *η*) parameterizes the CBCT system. In an ideal aligned system, (*θ*, *φ*, *η*) = (0, 0, 0).

**Figure 2:**
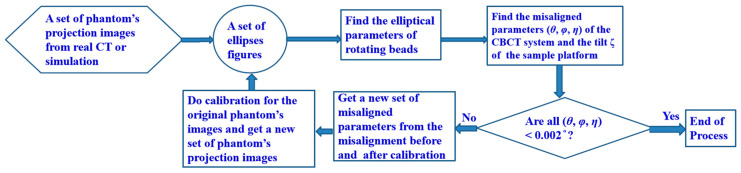
The PAL flowchart.

A misaligned system produces distorted images. Shih et al. [6] demonstrated that accurately determining the angles (*θ*, *φ*, *η*) can revive the fidelity of image. Our current approach varies from previous works (cited in Section 1) by using PAL’s iterative process, which starts with a dataset from either a simulation or an actual CT image to determine the angles (*θ*, *φ*, *η*). The essence of PAL is shown in Figure 2, but we modify it in this study to include finding the tilt angle ζ between the AOP and the AOR.

**Figure 3:**
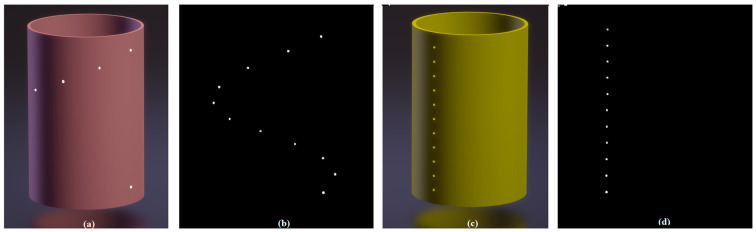
(**a**) The helical-beads phantom. (**b**) The inverted projection of the helical-beads phantom after removing the background around the beads. (**c**) The line-beads phantom. (**d**) The inverted projection of line-beads phantom after removing the background around the beads. Note: “The dots in (**b**,**d**) are the reversed projected images of steel beads”.

We calibrate the CBCT system using cylindrical phantoms: a helical-beads phantom (Figure 3a), and a line-beads phantom (Figure 3c). The helical-beads phantom is a cylinder with a 40 mm radius, containing eleven 2 mm diameter steel beads evenly spaced by 36° in azimuth and 10 mm in height. The line-beads phantom is a cylinder with a 40 mm radius, containing eleven 2 mm diameter steel beads evenly spaced by 10 mm along a line parallel to the AOP.

We acquired a set of projection images of a calibration phantom, inverted the images, and then used a method of threshold segmentation to remove each projection’s background around the beads (Figure 3b,d). Next, we applied the center-of-mass idea to the images to precisely calculate the exact beads’ coordinates.

### 2.2. Varied Rotational Radii of Beads in Phantoms

**Figure 4:**
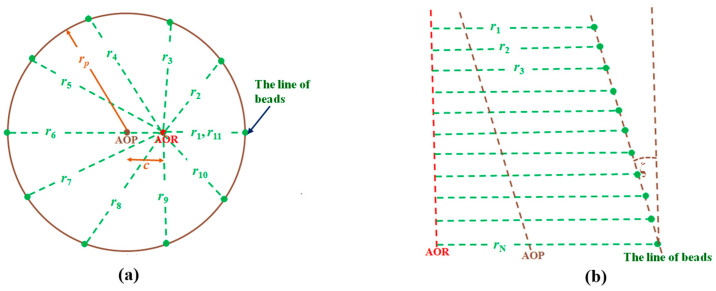
Two cases of varying radii of rotation. (**a**) When the helical-beads phantom’s AOP is parallel to the AOR but displaced. (**b**) When the line-beads phantom’s AOP has a tilt angle ζ from the AOR, where *r_i_* is the *i*^th^ bead’s rotational radius, *d* is two adjacent beads’ vertical separation, and *N* is the number of beads.

The beads on the two cylindrical phantoms shown in Figure 4 can have systematically varying radii of rotation under two conditions: (1) When the helical-beads phantom’s AOP is parallel to the AOR but displaced by a distance c in the x-z plane, as shown in Figure 4a; the beads’ rotating radii of this phantom are within the range r_p_-c to r_p_+c, where r_p_ is the radius of the phantom. (2) When there is a small tilt angle ζ between the line-beads phantom’s AOP and the AOR, the beads’ rotating radii vary, as shown in Figure 3b. The tilt angle ζ can be determined by Equation (1):

(1) $$\sin\zeta =\left|\frac{\left(r_{N}-r_{1}\right)}{(N-1)d}\right|$$

where *d* is the vertical separation of two adjacent beads, *N* is the number of beads, and *r*_1_, *r*_2_, *r*_3_, …, *r_N_* are the respective beads’ rotating radii of the line-beads phantom. Equation (1) also applies to the helical-beads phantom, since the first bead and the last beads are located along a line parallel to the AOP. To detect the tilt angle accurately, it is important to setup the AOR, AOP, and the line of beads within the same plane.

The actual vertical separation *d*’ must be modified according to Equation (2):

(2) d'=d×cosζ

### 2.3. The Ideal System

**Figure 5:**
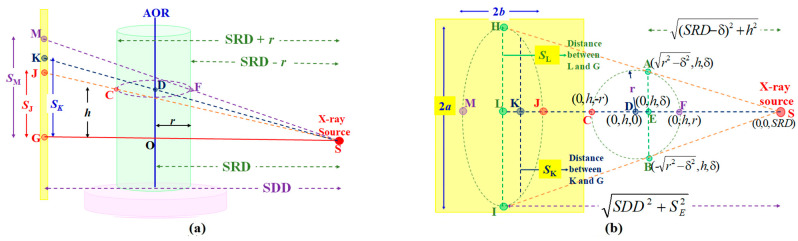
Two views of the projections on the detector (in yellow color) of the critical points on a circular track of radius *r* of a bead rotating about the AOR for an ideal system. (**a**) J and M on the detector are the respective projected positions of C and F on the bead’s circular track at *y* = *h*. K is the projection of D, the center of the circular track. (**b**) Critical points A and B on the bead’s circular track in the *x-z* plane have their projections H and I, respectively. The six critical points’ coordinates on the bead’s circular track are shown in the figure, where δ = *r*^2^/*SRD*.

As shown in our previous work [6], the projections of the rotating beads on the detector form ellipses with different eccentricities. In an ideal detector, *θ* = *φ* = *η* = 0. Figure 5 is a ray diagram showing points H, I, J, K, L, and M on the ideal detector (in yellow) as projections of the respective positions A, B, C, D, E, and F on a circular track traced by a bead on the rotating phantom. These six critical projected points are sufficient to derive the equations needed to yield the necessary set of elliptical parameters: (*U*_L_, *V*_L_), the elliptical center; *a* and *b*, the semi-major and semi-minor axes; and η*, the slant angle of the ellipse. In an ideal detector, η* = 0.

As shown in Figure 5a, two elliptical co-vertices J and M on the detector are the respective projected positions of C and F, therefore the vertical projections *S*_J_ and *S*_M_ are as Equation (3):

(3) $$S_{J}=h\frac{SDD}{SRD+r};\;\;\;\;\;\;\;S_{M}=h\frac{SDD}{SRD-r}$$

where *S*_J_ and *S*_M_ are the distances from M and J (the co-vertices) to the center G (Figure 5a), respectively, *r* the bead’s rotating radius, and *h* is the distance from the rotating bead to the *z*-axis.

The system’s magnification factor at D, the center of bead’s circular track, *M* is related to the position of K, the projection of D on the detector:

(4) $$M=\frac{S_{K}}{h}=\frac{SDD}{SRD};\;\;\;\;\;\;S_{K}=\frac{SDD}{SRD}h=M\times h\;$$

where *S*_K_ is the distance from K to the center G, as shown in Figure 5a.

The magnification factor *M*′, at the critical positions of A, B, and E, is related to their respective projections at H, I, and L on the detector, as shown in Figure 5b:

(5) $$M\prime =\frac{SDD}{SRD-\delta }=\frac{S_{L}}{h}=\frac{a}{\sqrt{r^{2}-\delta^{2}}}=\frac{SRD^{2}}{SRD^{2}-r^{2}}\;\frac{SDD}{SRD}$$

where δ =*r*^2^/*SRD*, and *S*_L_ is the distance from the elliptical center L to the center G.

(6) $$S_{L}=\frac{\left(S_{M}+S_{J}\right)}{2}=\left(\frac{SRD^{2}}{SRD^{2}-r^{2}}\frac{SDD}{SRD}\right)h=M\prime \times h$$

The semi-major axis *a* and the rotating radius *r* are related according to Equation (7):

(7) $$a=r\frac{SDD}{\sqrt{SRD^{2}-r^{2}}};\;\;\;\;\;\;r=a\frac{SRD}{\sqrt{SDD^{2}+a^{2}}}$$

Since the beads’ rotating radii vary, the projected ellipses on the detector have different semi-major axes.

For the projected ellipse on the detector, the relationships between ‘*b*’ (the semi-minor axis), ‘*h*’ (the rotating bead’s height) ‘*r*’, (the bead’s rotating radius), and ‘*S*_L_’ (the distance between L, the center of the ellipse, and the center G), are described by Equation (8).

(8) $$b=\frac{\left(S_{M}-S_{J}\right)}{2}=\left(\frac{SRD^{2}}{SRD^{2}-r^{2}}\frac{SDD}{SRD}\frac{r}{SRD}\right)h=S_{L}\frac{r}{SRD};\;\;\;\;\;\;S_{L}=\frac{b}{r}SRD$$

Equation (8) provides *S*_L_, the projected elliptical centers of the beads, as a function of *b/r*, semi-minor axis divides by respective bead’s rotating radius, and *S*_L_ = *S*_L_(*b/r*). The *S*_L_ vs. *b/r* plot for the elliptical centers above and below the center G determines V_G._ Since all the projected ellipse centers constitute a vertical line, we acquire the center G, (*U_G_*, *V_G_*). After finding the center G, *S*_K_ can be calculated using Equation (4).

If a rotating bead is on the system’s principal axis (z-axis), i.e., *h* = 0, then on the detector, *b* = 0, and *S_L_* = 0; its projection is a horizontal line of length 2*a* passing through the center G, a crucial point on the detector. However, even without a bead at *h* = 0, we can still find G by considering the following: If we plot all projected ellipses on the *U-V* plane, the ellipses flatten as the corresponding circular track’s *h* approaches 0, as expected. This suggests that in a *V_L_* vs. *S_L_* plot, the ellipses will form points to which two straight lines with opposite slopes can be fitted, intersecting at *V* for G. This will be shown in the calibration results in Section 4.

As the rotating bead approaches the *z*-axis, *b*→0 on the detector. The elliptical parameters (*U_L_*, *V_L_*, *a*, *b*, η*) will be determined by linear interpolation rather than fitting an ellipse.

In an ideal detector, the vertical projected distance *S*_d_ between two adjacent beads’ rotating centers is the same, i.e.,

(9) $$S_{d}=Md\prime$$

### 2.4. The Misaligned System

We consider the case where *η*, *φ*, and *θ* ≤ 10°, then sin*η*~*η*, sin*φ*~*φ*, sin*θ*~*θ*, and cos*η*~cos*φ*~cos*θ*~1. After neglecting the terms of second-order, the simplified matrix of transformation *T* for a misaligned system is:

(10) $$T=\left[\begin{array}{ccc} 1 & 0 & 0\\ 0 & \cos\theta & -\sin\theta \\ 0 & \sin\theta & \cos\theta \end{array}\right]\left[\begin{array}{ccc} \cos\varphi & 0 & \sin\varphi \\ 0 & 1 & 0\\ -\sin\varphi & 0 & \cos\varphi \end{array}\right]\left[\begin{array}{ccc} \cos\eta & \sin\eta & 0\\ -\sin\eta & \cos\eta & 0\\ 0 & 0 & 1 \end{array}\right]\approx \left[\begin{array}{ccc} 1 & \eta & \varphi \\ -\eta & 1 & -\theta \\ -\varphi & \theta & 1 \end{array}\right]$$

The point (U_0_, V_0_, 0) in an aligned *U*′–*V*′–*W*′ local system transforms to (U_0_+ V_0_*η*, –U_0_*η* + V_0_, –U_0_*φ* + V_0_*θ*) in a misaligned detector, relative to the original coordinate system. (U_0_ + V_0_*η*, –U_0_*η* + V_0_, –RDD –U_0_*φ* + V_0_*θ*) are their respective global Cartesian coordinates, where RDD = SDD − SRD. The small *z* coordinate’s variation causes variations in the magnification factor across the four quadrants on a misaligned detector.

*M_l_* and *M_u_* are the respective magnification factors of the beads below and above *z*-axis:

(11) $$M_{l}=M(1-\frac{h_{l}\times \theta }{SRD});\;\;\;\;\;\;\;M_{u}=M(1-\frac{h_{u}\times \theta }{SRD})$$

where *h_l_* (*h_l_* < 0) and *h_u_* are the bead farthest below and above *z*-axis, respectively. Using Equation (9), the average difference ΔS_d_ in the vertical projected separation between two adjacent beads’ rotating centers below and above the *z*-axis is:

(12) $$\Delta S_{d}=(M_{u}-M_{l})\;\times \;d’\;=\frac{M(h_{u}-h_{l})\times d'\times \theta }{SRD}=\frac{S_{d}\times (phantoms’\;height)\times \theta }{SRD}$$

(13) $$\theta =\frac{\Delta S_{d}}{S_{d}}\times \frac{SRD}{(\mathrm{phantom}’s\;\mathrm{height})}$$

where *N* is the total bead number, (*N* − 1) × *d′* ≤ (*h_u_* − *h_l_*) ≤ (*N* + 1) × *d’*, and (*h_u_* − *h_l_*) ≈ phantoms height. The detector tilt angle *θ* is obtained from Equation (13). *N* × *d′* is set as the phantom’s height since the calculated *θ* value and the setting value of the model are closely matched. Equation (13) then acquires the tilt angle *θ*. 

In a misaligned detector, the bead positions A and B have distinct magnification factors due to the tilt angle *θ* and skew angle *φ*. The positions H and I are their respective projections on the detector. Let *M*_A_ and *M*_B_ be the magnification factors for the beads’ respective positions of A and B, then:

(14) $$M_{A}=\frac{\left[SDD+a\varphi -S_{L}\theta \right]}{SRD-\delta };\;\;\;\;\;\;\;M_{B}=\frac{\left[SDD-a\varphi -S_{L}\theta \right]}{SRD-\delta }$$

For *η* = 0, *θ* ≠ 0, and *φ* ≠ 0, the two projected elliptical vertex points H and I on the detector are:

(15) $$\left(U_{H},\;V_{H})=(M_{A}\sqrt{r^{2}-\delta^{2}},M_{A}h);\;\;\;\;\;\;\;(U_{I},\;V_{I})=(-M_{B}\sqrt{r^{2}-\delta^{2}},M_{B}h\right)$$

(16) $$\eta *\approx \tan\eta *=\frac{\left(V_{H}-V_{I}\right)}{\left(U_{H}-U_{I}\right)}=\frac{(M_{A}-M_{B})h}{(M_{A}+M_{B})\sqrt{r^{2}-\delta^{2}}}\approx \frac{h}{SRD-\delta }\varphi =\frac{S_{L}}{SDD}\varphi$$

A skew angle *φ* in a misaligned detector will cause a slant angle η* = *φ* × *S_L_*/*SDD* for the projected ellipse. If a rotating bead is on the system’s principal axis, it traces a horizontal line on the detector, so η* = 0. If *η* ≠ 0, then *η* represents the rotational angle about “*W′*-axis” (normal to the plane of detector), therefore making η* = *η* the center G. The slant angle η* in Equation (16) requires adding “*η*” to the original η* in each ellipse:

(17) $$\eta *\approx \eta +\frac{S_{L}}{SDD}\varphi$$

which shows η* is linear with *S_L_* and detector’s skew angle *φ*. The slant angles η* of all projected ellipses are plotted against ‘*S*_L_’ of each respective bead to determine the detector’s skew angle *φ* from the plot’s slope and the slant angle *η* at the center G, given by Equation (17).

Since a first-order angular approximation is used, there is larger difference between the calculated misalignment and the actual data values (>10°). The PAL effectively reduces these differences.

The misaligned system is calibrated using the resulting angles (*θ*, *φ*, *η*) to generate a new set of near-ideal detector images. Afterward, we determine the center G (*U*_G_, *V*_G_), the CBCT system’s magnification factor M, and SRD is recalibrated from Equation (4), while SDD is measured from the CBCT system directly.

## 3. Validations

### 3.1. The Criterion for the Validation of Analytical Simulation

PAL’s ability to detect misalignment and restore alignment was tested on a detector of a dental CBCT with 2176 × 1792 pixels and a pixel size of 0.139 mm in both dimensions. The SRD and SDD were set at 430 mm and 620 mm, respectively, and SRD was re-acquired using Equation (4). The inputs and results of these tests are presented in Section 3.2 and Section 3.3 and summarized in Table 1 and Table 2. The input setting values of calibrated parameters are shown in the row labeled “Model”. In the row labeled “Found”, the misalignment detected by PAL’s iterative process is presented. PAL can accurately determine all the misalignment angles, SRD, and the geometric center (*U*_G_, *V*_G_) at once. Alignment is achieved when (*θ*, *φ*, *η*) < 0.002° (3 × 10^−^^4^ rad) after calibration, as listed in the row labeled “Aligned”. For this setup, all misaligned angles are <0.002° after calibration.

### 3.2. The Helical-Beads Phantom’s AOP Has a Tilt Angle ζ from the AOR

First, the helical-beads phantom (Figure 3a) was set with its AOP parallel to the AOR but offset by *c* = 10.0 mm (Figure 4a). Then, the AOP is tilted by a small angle ζ. The rotating radii of the beads were set from 30.0 mm to (50.0 + 100.0 × sinζ) mm for all tests. The results of nine analytical simulations are shown in Table 1. The errors in SRD are within 0.1%, and that in (*U*_G_, *V*_G_) is within 0.5 pixels of the set values. The errors of the misaligned angles (*θ*, *φ*, *η*) are within ±0.03° of their set values, respectively.

### 3.3. The Line-Beads Phantom’s AOP Has a Tilt Angle ζ from the AOR

The line-beads phantom is shown in Figure 3c, with its AOP having a small tilt angle ζ from the AOR. The rotating radii of the beads (Figure 4b) were set from 40.0 mm to (40.0 + 100.0 × sinζ) mm for all simulations. The results of nine analytical simulations are shown in Table 2. The errors in SRD are within 0.1%, and the errors in (*U*_G_, *V*_G_) are within 0.5 pixels of the set values. The errors in the misalignment angles (*θ*, *φ*, *η*) are within ±0.03° of their set values, respectively.

From the simulated tests in Table 1 and Table 2, we find that the two sets of results are almost identical, with negligible differences. We conclude that the calibration results are independent of the choice of those two phantoms. The error in the tilt angle ζ between the AOP and the AOR is within ±0.004°of the set values for ζ ≤ 10°. The error in angle ζ is increasing as ζ increasing, and this error is around 0.04%.

## 4. Results

### 4.1. Our Dental CBCT System

Having validated PAL’s efficacy, we used it to calibrate our home-made dental CBCT system with the same geometry as in Section 3. Ellipses on the detector are traced by the phantom’s rotating beads, forming PAL’s input data. The output of the final result is the system free from misalignment after calibration, i.e., (*θ*, *φ*, *η*) ≈ (0, 0, 0). With variances < 0.002° (3 × 10^−^^5^ rad).

### 4.2. Calibrating Our Dental CBCT System

Table 3 lists the elliptical center (*U*_L_, *V*_L_), semi-axes *a* and *b* (in pixels), slant angle η* for each ellipse, *r* (each bead’s rotating radius), and the value of (*b*/*r*) × SRD, which represents the projected distance of the elliptical center from the geometric center G. V_K_ is the projected distance of the beads from the principal axis, all derived from the CBCT image by PAL’s handling of the calibration phantom, thus “before calibration”. In Table 3 and Table 4, the varying bead rotating radii *r* confirms the AOP has a small tilt from the AOR.

**Figure 6:**
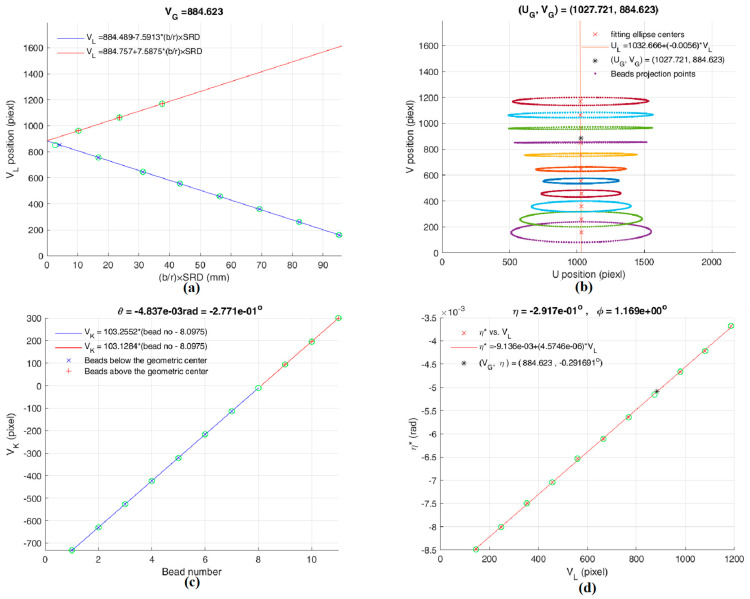
Primary calculated parameters of our dental CBCT system before calibration. (**a**) Ellipse centers position V_L_ vs. (*b/r*) × SRD. (**b**) Elliptical centers in *V-U* coordinates. (**c**) Projected rotational centers V_K_ vs. bead number (above and below the *z*-axis). (**d**) Elliptical skew angle η* vs. V_L_.

**Figure 7:**
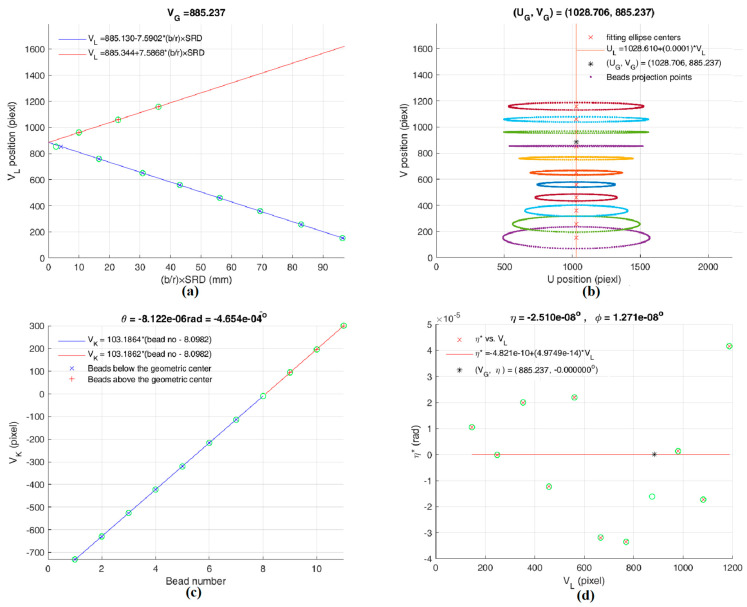
The final calculated parameters of our dental CBCT system after calibration. (**a**) Ellipse center’s position V_L_ vs. (*b*/*r*) × SRD. (**b**) Elliptical centers in *V-U* coordinates. (**c**) Projected rotational centers V_K_ vs. the bead number (above and below the *z*-axis). (**d**) Elliptical skew angle η* vs. V_L_.

Figure 6 is derived from Table 3. Figure 6a,b yield (*U*_G_, *V*_G_); Figure 6c and Equation (13) yield *θ* = −0.277°; Figure 6d and Equation (17) yield *φ* = 1.169° and *η* = −0.292°. Thus, (*θ*, *φ*, *η*) = (−0.277°, 1.169°, −0.292°) is the misalignment from the primary calculation before calibration. Using PAL, (*θ*, *φ*, *η*) = (−0.231°, 1.152°, −0.291°) is the misalignment from the final calculation. After calibration, we converted the misaligned parameters into “ideal aligned parameters,” and the final results are shown in Table 4 and Figure 7. From Table 4, the tilt angle ζ between the AOR and AOP can be determined as:

ζ = sin^−^^1^ |(51.821 − 53.646)/[(11 − 1) × 10)]| = 1.046° (±0.001°).

After calibration, the geometric center G is (*U*_G_, *V*_G_) = (1028.706, 885.237) pixels, and the CBCT magnification factor *M* is 1.4328 (from Equation (4)), therefore the SRD is 432.474 mm; Equation (13) gives *θ*=(−5 × 10^−4^)°, Equation (17) gives *φ*=(1 × 10^−^^8^)°, and *η*=(−3 × 10^−^^8^)°. PAL effectively reduces the detector’s misalignment from (*θ*, *φ*, *η*) = (−0.231°, 1.152°, −0.291°) before calibration to (*θ*, *φ*, *η*) = ((−5 × 10^−^^4^)°, (1 × 10^−^^8^)°, (−3 × 10^−^^8^)°) after calibration. The slant angles η* of all ellipses are reduced to <4 × 10^−^^5^ rad (0.003°). The CBCT system reached an ideal alignment within ±0.001° in all three directions after calibration.

### 4.3. Calibrating a Dental CBCT System with Analytical Simulation

An imitation CBCT system, similar to our dental CBCT, was calibrated for validation. The results of simulations are shown in Table 5. The errors in SRD are within 0.01%, and the errors in (*U*_G_, *V*_G_) are within 0.02 pixels of the set values. The errors in the misalignment angles (*θ*, *φ*, *η*) are within ±0.001° of the relevant set values. The error of the tilt angle ζ between the AOP and the AOR is within ±0.001°of the set value. The errors of measurement uncertainty in a real CBCT system is not included.

### 4.4. The Reconstructed Images of the HA Phantom

**Figure 8:**
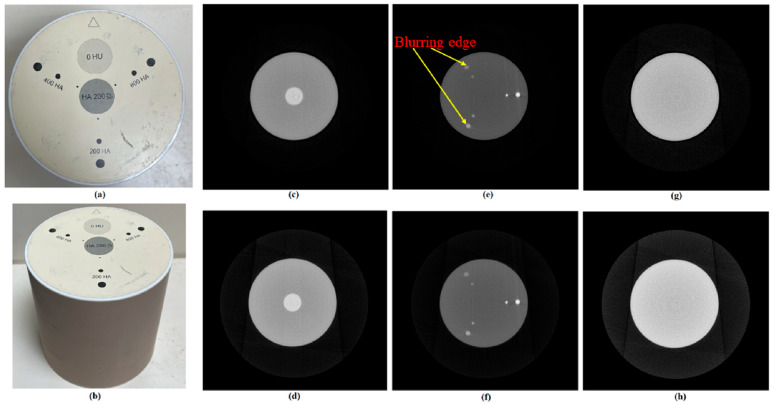
The HA phantom and its reconstructed images. (**a**) Top view of the HA phantom. (**b**) Side view of the HA phantom. (**c**)Reconstructed image of slice number 365 without calibration (CS region). (**d**) Reconstructed image of slice number 365 with calibration (CS region). (**e**) Reconstructed image of slice number 775 without calibration (HA region). (**f**) Reconstructed image of slice number 775 with calibration (HA region). (**g**) Reconstructed image of slice number 850 without calibration (water region). (**h**) Reconstructed image of slice number 850 with calibration (water region).

The HA phantom was designed to analyze CT reconstruction quality. The reconstructed image of the HA phantom is shown in Figure 8. There is blurring circular edge in the reconstructed image without calibration. We performed SNR and CNR analysis for the image in the CS region (slice number 365). Both values improved after calibration. The results are shown in Table 6 and Table 7.

## 5. Discussions and Conclusions

CBCT is becoming increasingly important in treatment planning and diagnosis in fields such as dental implantology, otolaryngology, orthopedics, and interventional radiology (IR). Due to the increasing use of this technology, CBCT scanners now have many applications in dentistry, including oral surgery, endodontics, and orthodontics. Integrated CBCT is also an important tool for patient positioning and verification in image-guided radiotherapy (IGRT). The quality of CBCT images depends on accurate knowledge of the CBCT system alignment. A misaligned system produces blurred and distorted images of reconstruction. Our novel calibration procedure accurately determines misalignment angles with high precision and can revive the fidelity of image, enhancing potential clinical applications.

All commercially available C-arm CBCT equipment uses digital flat-panel detectors. C-arm CBCT allows the acquisition of volumetric data in a single rotation of the source and detector. The implementation of CBCT on a mobile C-arm for 3D intraoperative guidance has recently been studied. Our novel calibration procedure can be used in the pre-calibration (offline calibration) of a C-arm system.

Yang et al. [3] gave the errors of misaligned angles (*θ*, *φ*, *η*) within 0.04° and 0.1% error in SDD, but needed precisely set beads’ locations and configurations, and the AOP and the AOR to overlap. Our previous work [6] required only that the two axes be parallel but not overlap, and the beads’ rotating radius be > 10mm.

In this paper, we have revised and validated PAL, our precise calibrating algorithm, by using analytical simulations for the setting models and calibrating a CBCT system with PAL using cylindrical phantoms of a helical-beads (Section 3.2) and a line-beads (Section 3.3), under three conditions: (1) the detector’s misaligned angles (*θ*, *φ*, *η*) be each <10°, to fit the first-order approximation of the trigonometric functions in Equation (10), the transformation matrix *T*; (2) all critical knowledge of misalignment comes from the parameters describing the ellipses projected on the detector by the beads with varied rotation of radii about the AOR; and (3) the AOP need not be parallel to the AOR, but any small tilt angle ζ between the two axes can be determined.

We found our new procedure to: (1) calibrate the CBCT system more precisely than our previous work [6] regarding the errors of misaligned angles (*θ*, *φ*, *η*); (2) determine the tilt angle between the AOP and AOR when it is not 0°; and (3) work equally well with different phantoms. We conclude that our generalized procedure presented above can give a complete, precise characterization of any CBCT system, including a tilting sample platform.

## Figures and Tables

**Table 1:** Validation of analytical simulations when the helical-beads phantom’s AOP has a tilt angle ζ from the AOR.

Simulating Setups	U_G_ (Pixel)	V_G_ (Pixel)	SRD (mm)	*θ* (^o^)	*φ*(^o^)	*η*(^o^)	*ζ*(^o^)	Verification
1	1000.000	800.000	430.000	0.000	0.000	10.000	2.000	Model
	984.667	816.740	436.625	0.000	0.000	10.000	2.031	Found
	1000.000	800.000	430.000	0.000	0.000	0.000	2.000	Aligned
2	1000.000	900.000	430.000	0.000	10.000	0.000	3.000	Model
	991.696	899.983	429.982	0.000	10.000	0.001	3.049	Found
	999.700	900.014	430.017	0.000	0.000	0.000	3.000	Aligned
3	1000.000	1000.000	430.000	10.000	0.000	10.000	4.000	Model
	1019.396	1015.517	427.668	10.008	0.000	10.000	4.776	Found
	999.632	1000.276	430.625	0.000	0.000	0.000	4.003	Aligned
4	1100.000	800.000	430.000	1.000	1.000	1.000	5.000	Model
	1097.653	799.806	429.777	1.000	1.000	1.000	5.079	Found
	1099.994	800.041	430.002	0.000	0.000	0.000	5.000	Aligned
5	1100.000	900.000	430.000	2.000	2.000	2.000	6.000	Model
	1098.800	899.642	429.527	2.001	2.000	2.000	6.164	Found
	1099.999	900.000	429.997	0.000	0.000	0.000	6.000	Aligned
6	1100.000	1000.000	430.000	3.000	3.000	3.000	7.000	Model
	1103.442	999.502	429.243	3.004	3.000	3.000	7.255	Found
	1100.008	1000.120	429.970	0.000	0.000	0.000	7.000	Aligned
7	1200.000	800.000	430.000	4.000	4.000	4.000	8.000	Model
	1190.606	792.391	428.921	4.007	4.000	4.009	8.353	Found
	1199.634	800.297	429.930	0.000	0.001	-0.001	7.998	Aligned
8	1200.000	900.000	430.000	5.000	5.000	5.000	9.000	Model
	1196.989	890.534	428.554	5.016	5.000	5.011	9.459	Found
	1199.790	899.993	429.838	0.000	0.000	0.000	8.999	Aligned
9	1200.000	1000.000	430.000	6.000	6.000	6.000	10.000	Model
	1206.877	988.686	428.136	6.025	5.999	6.014	10.573	Found
	1200.018	1000.059	429.687	0.000	0.000	0.000	10.001	Aligned

**Table 2:** Validation of the analytical simulations when the line-beads phantom’s AOP has a tilt angle ζ from the AOR.

Simulating Setups	U_G_ (Pixel)	V_G_ (Pixel)	SRD (mm)	*θ* (^o^)	*φ*(^o^)	*η*(^o^)	*ζ*(^o^)	Verification
1	1000.000	800.000	430.000	0.000	0.000	10.000	2.000	Model
	984.667	816.740	436.625	0.000	0.000	10.000	2.031	Found
	1000.000	800.000	430.000	0.000	0.000	0.000	2.000	Aligned
2	1000.000	900.000	430.000	0.000	10.000	0.000	3.000	Model
	990.802	899.980	429.983	0.000	10.000	0.001	3.050	Found
	999.700	900.014	430.017	0.000	0.000	0.000	3.000	Aligned
3	1000.000	1000.000	430.000	10.000	0.000	10.000	4.000	Model
	1019.396	1015.517	427.520	10.008	0.001	10.000	5.010	Found
	999.632	1000.276	430.623	0.000	0.000	0.000	4.003	Aligned
4	1100.000	800.000	430.000	1.000	1.000	1.000	5.000	Model
	1097.499	799.805	429.762	1.000	1.000	1.000	5.102	Found
	1099.994	800.041	430.002	0.000	0.000	0.000	5.000	Aligned
5	1100.000	900.000	430.000	2.000	2.000	2.000	6.000	Model
	1098.425	899.639	429.486	2.001	2.000	2.000	6.210	Found
	1099.999	900.000	429.997	0.000	0.000	0.000	6.000	Aligned
6	1100.000	1000.000	430.000	3.000	3.000	3.000	7.000	Model
	1102.779	999.507	429.169	3.004	3.000	3.000	7.324	Found
	1100.008	1000.120	429.970	0.000	0.000	0.000	7.000	Aligned
7	1200.000	800.000	430.000	4.000	4.000	4.000	8.000	Model
	1189.585	792.419	428.804	4.007	4.000	4.008	8.444	Found
	1199.634	800.297	429.930	0.000	0.001	-0.001	7.998	Aligned
8	1200.000	900.000	430.000	5.000	5.000	5.000	9.000	Model
	1195.542	890.607	428.384	5.016	5.000	5.011	9.573	Found
	1199.790	899.993	429.839	0.000	0.000	0.000	8.999	Aligned
9	1200.000	1000.000	430.000	6.000	6.000	6.000	10.000	Model
	1204.934	988.834	427.900	6.025	5.999	6.014	10.710	Found
	1200.018	1000.059	429.687	0.000	0.000	0.000	10.001	Aligned

**Table 3:** The elliptical parameters of each bead for our dental CBCT system before calibration.

Bead No.	*U*_L_ (Pixel)	*V*_L_ (Pixel)	Semi-Major Axis (*a*)	Semi-Minor Axis (*b*)	η* (Rad)	*r*(mm)	(*b/r*) × *SRD* (mm)	*V*_K_ (Pixel)
1	1031.2101	160.3275	519.8593	79.3203	−0.00849	51.8307	97.3269	−731.5368
2	1031.0564	260.2956	451.6038	59.6192	−0.00801	45.0998	83.8444	−630.1795
3	1030.7361	359.1330	367.5386	40.9339	−0.00749	36.7676	70.1228	−526.6523
4	1030.4389	457.8673	294.6665	26.7232	−0.00704	29.5132	56.3831	−423.1621
5	1030.0572	555.1430	281.1494	19.6407	−0.00653	28.1648	42.7870	−321.1075
6	1029.5691	646.3118	333.6322	16.7880	−0.00611	33.3955	28.8167	−216.1962
7	1028.6118	756.4342	414.7303	11.2045	−0.00564	41.4503	15.2588	−114.1042
8	1026.8056	852.3691	489.3404	3.3211	−0.00515	48.8252	1.3589	−9.9114
9	1027.8958	962.8579	534.0376	8.8082	−0.00466	53.2246	12.4251	94.2877
10	1026.5521	1064.1786	537.0056	20.3251	−0.00422	53.5162	25.8672	195.0878
11	1025.8983	1169.7566	503.9358	30.3415	−0.00368	50.2634	39.3870	299.6775

**Table 4:** The elliptical parameters of each bead for our dental CBCT system after calibration.

Bead No.	*U*_L_ (Pixel)	*V*_L_ (Pixel)	Semi−Major Axis (*a*)	Semi−Minor Axis (*b*)	η* (Rad)	*R* (mm)	(*b/r*) × *SRD* (mm)	*V*_K_ (Pixel)
1	1028.6660	152.4467	539.6596	83.2825	0.00001	53.6460	97.2815	−731.0551
2	1028.7245	257.0081	466.3374	61.9410	0.00000	45.8087	83.8129	−629.8214
3	1028.5931	359.2661	377.5675	42.0892	0.00002	36.1071	70.1027	−526.3997
4	1028.5849	460.3167	301.1520	27.1958	−0.00001	27.6604	56.3723	−422.9981
5	1028.6759	558.8318	285.8904	19.7872	0.00002	26.0904	42.7827	−321.0099
6	1028.8164	650.2557	337.6639	16.7547	−0.00003	32.1808	28.8166	−216.1482
7	1028.7173	759.5510	417.3701	11.0565	−0.00003	41.5594	15.2604	−114.0851
8	1027.7668	853.7684	489.5930	3.2483	−0.00002	50.1466	1.3614	−9.8960
9	1029.6001	961.1178	531.7960	8.5112	0.00000	55.2690	12.4292	94.2989
10	1028.8055	1058.5057	532.0376	19.4388	−0.00002	55.6084	25.8771	195.1268
11	1028.5440	1158.9218	496.6324	28.7115	0.00004	51.8211	39.4054	299.7656

**Table 5:** Validation of the analytical simulation for our dental CBCT system.

Simulating Setups	U_G_ (Pixel)	V_G_ (Pixel)	SRD (mm)	*θ*(^o^)	*φ*(^o^)	*η*(^o^)	*ζ*(^o^)	Verification
1	1029.000	885.000	432.000	−0.230	1.150	−0.290	1.046	Model
	1028.193	884.699	432.038	−0.230	1.150	−0.290	1.072	Found
	1028.984	884.997	432.000	−2 × 10^−5^	1 × 10^−4^	−1 × 10^−4^	1.046	Aligned

**Table 6:** The SNR and CNR for the reconstructed image without calibration of slice no. 365.

	Center	Up	Down	Left	Right
SNR	5.4567	4.4587	4.4811	4.4994	4.4583
CNR	/	1.3055	1.3055	1.2839	1.2846

**Table 7:** The SNR and CNR for the reconstructed image with calibration of slice no. 365.

	Center	Up	Down	Left	Right
SNR	7.0657	6.8115	6.7803	6.8228	6.8339
CNR	/	1.5661	1.5603	1.5449	1.5483

## Data Availability

Data available on request from the authors.
